# Supplementary material for: The Shared Proteome of the Apomictic Fern Dryopteris affinis ssp. affinis and Its Sexual Relative Dryopteris oreades
Source: Int J Mol Sci. 2022 Nov 14;23(22):14027. doi: 10.3390/ijms232214027 (PMC9693225; doi:10.3390/ijms232214027)
Supplement: Supplementary file 1 [file ijms-23-14027-s001.zip › ijms-1987885-Table S2. Only_String interaction's strength.pdf]

Table S2. Functional STRING interactions of proteins extracted from gametophytes of *Dryopteris affinis* and *D. oreades*, which were grouped as growth and reproduction, phytohomones, gene expression, and abiotic response.

Node1	Node2	Neighborhood	Co-occurrence	Homology	Co-expression	Experiments	Database	Textmining	Score
				Growth-Reproduction					
ENO1	GAPCP-1	0.184	0	0	0.869	0.678	0	0.544	0.982
ENO1	GAPCP-2	0.184	0	0	0.805	0.678	0	0.550	0.973
ENO1	PGM2	0	0	0	0.901	0.217	0	0.625	0.968
ENO1	PGMP	0	0	0	0.901	0.217	0	0.567	0.963
PGM2	PGMP	0	0.449	0.954	0.085	0	0.800	0.645	0.818
PUR4	SHM4	0.174	0	0	0.521	0	0	0.577	0.818
ACT7	PRF5	0	0	0	0.045	0.512	0.390	0.373	0.798
CPN60A	SHD	0	0	0	0.312	0.185	0	0.667	0.797
EMB2761	OVA4	0.190	0	0	0.272	0	0	0.647	0.773
ACT7	VLN2	0	0	0	0.046	0.184	0.612	0.330	0.770
OVA4	OVA9	0.190	0	0	0.128	0	0	0.694	0.764
OVA9	PRP8A	0	0	0	0.292	0.053	0	0.664	0.755
AT3G03960	CPN60A	0	0	0	0.365	0.311	0.301	0.189	0.718
EMB2761	OVA9	0.162	0	0	0.240	0.046	0	0.591	0.718
SHD	SQN	0	0	0	0.112	0.182	0.257	0.539	0.717
				Phytohormones					
IBR1	PKT3	0.058	0	0	0.141	0.063	0.293	0.649	0.778
ARFA1F	AT3G08530	0	0	0	0.042	0.167	0.494	0.477	0.760
				Gene expression					
AT1G57660	AT3G60245	0.190	0.202	0	0.934	0.853	0.656	0.546	0.998
AT1G09640	HOG1	0	0	0	0.923	0	0	0.218	0.937
AT1G09640	UAP56a	0	0	0	0.915	0.225	0	0.148	0.939
AT1G09640	AT3G60245	0	0	0	0.914	0	0	0	0.914

RIN1	UAP56a	0	0	0	0.910	0.094	0	0.055	0.916
AT1G09640	AT1G57660	0	0	0	0.903	0.676	0	0	0.967
AT1G09640	AT2G40290	0	0	0	0.855	0.047	0	0.599	0.939
AT1G09640	EIF4A1	0	0	0	0.843	0.225	0	0.636	0.952
AT5G08650	SCO1	0	0.407	0.572	0.841	0	0.308	0.511	0.924
HTA9	UAP56a	0	0	0	0.839	0.060	0.156	0.257	0.892
AT1G09640	SCO1	0	0	0	0.792	0.750	0	0.545	0.974
AT1G57660	AT2G40290	0	0	0	0.785	0	0	0.330	0.850
AT2G40290	EIF4A1	0	0	0	0.736	0.554	0.541	0.546	0.972
AT1G09200	HTB1	0	0	0	0.463	0.379	0.389	0.380	0.856
HTB1	T11P11.4	0	0	0	0.263	0.491	0.391	0.372	0.837
AT1G09640	RIN1	0	0	0	0.240	0.142	0	0.607	0.721
HTA9	HTB1	0	0	0	0.230	0.646	0.451	0.354	0.890
AT1G09200	T11P11.4	0	0	0	0.199	0.435	0.284	0.804	0.928
HTA9	T11P11.4	0	0	0	0.133	0.677	0.452	0.627	0.935
AT1G09200	HTA9	0	0	0	0.128	0.524	0.448	0.516	0.874
HTA9	RIN1	0	0	0	0.125	0.873	0.644	0.712	0.987
AT2G40290	AT2G42520	0	0	0	0.108	0.226	0.374	0.875	0.939
AT1G09640	HTA9	0	0	0	0.099	0	0	0.712	0.729
HTB1	RIN1	0	0	0	0.045	0.753	0.437	0.076	0.860
AT1G09200	RIN1	0	0	0	0	0.677	0.462	0.157	0.840
RIN1	T11P11.4	0	0	0	0	0.764	0.254	0.106	0.828
					Abiotic stress				
AOR	PRXQ	0.102	0	0	0.941	0	0	0	0.944
AT3G60750	TRA2	0.190	0	0	0.774	0.720	0.966	0.710	0.999
CSD1	MSD1	0	0	0	0.116	0.722	0.902	0.928	0.998
Hsp70-15	Hsp81.4	0	0	0	0.794	0.620	0.380	0.662	0.981
CAT	CSD1	0.172	0	0	0.126	0.445	0.685	0.847	0.977
ELF5A-3	RPL10B	0	0	0	0.902	0.762	0	0.078	0.976
CAT	GOX1	0	0	0	0.213	0	0.923	0.575	0.972
CR88	Hsp70-15	0	0	0	0.520	0.620	0.380	0.765	0.969
AT3G29320	SEX1	0.190	0	0	0.863	0	0	0.732	0.967
At5g06290	TRX-M4	0.058	0	0	0.339	0.219	0.638	0.805	0.959
PRXQ	TRX-M4	0	0	0	0.684	0	0.501	0.741	0.955

At5g06290	CAT	0.067	0	0	0.601	0.520	0	0.770	0.953
Hsp81.4	MTHSC70-2	0	0	0	0.575	0.220	0.380	0.778	0.948
HSP91	Hsp81.4	0	0	0	0.462	0.620	0.380	0.641	0.948
CR88	HSP91	0	0	0	0.423	0.620	0.380	0.641	0.944
Hsp70-15	MTHSC70-2	0	0.412	0.713	0.821	0.184	0.480	0.672	0.940
CAT	MSD1	0.057	0	0	0.047	0.182	0.584	0.804	0.929
At5g06290	PRXQ	0.190	0.295	0.743	0.856	0.182	0	0.972	0.927
MTHSC70-2	ROF1	0	0	0	0.828	0.149	0.251	0.405	0.926
CAT	GPX7	0	0	0	0.205	0.439	0	0.840	0.922
GSTF10	GSTU19	0	0	0	0	0.045	0.263	0.894	0.919
GSTF9	GSTU19	0	0	0	0.074	0.045	0.263	0.870	0.903
CPN10	CR88	0	0	0	0.586	0.184	0	0.699	0.889
AT1G53540	HSP17.6II	0	0.448	0.849	0.863	0	0	0.870	0.888
AT3G52990	AT3G60750	0.110	0	0	0.141	0.048	0.600	0.668	0.885
CR88	MTHSC70-2	0	0	0	0.388	0.220	0.380	0.652	0.883
CAT	TRA2	0	0	0	0.752	0.357	0	0.325	0.882
AT3G60750	AT5G63680	0.110	0	0	0.130	0.048	0.600	0.663	0.882
DHAR3	GPX7	0	0	0	0.073	0	0.650	0.643	0.874
At5g06290	GPX7	0	0	0	0.043	0.258	0	0.827	0.867
AT2G22360	MTHSC70-2	0.042	0.434	0	0.195	0.480	0.254	0.310	0.856
ALDH5F1	AT5G14590	0.065	0	0	0.043	0	0.800	0.230	0.843
CSD1	NDPK1	0	0	0	0.114	0.659	0	0.511	0.839
ALDH5F1	cICDH	0.065	0	0	0.043	0	0.800	0.175	0.832
PRXQ	SHM1	0.071	0	0	0.826	0	0	0	0.831
At5g06290	DHAR3	0	0	0	0.430	0	0	0.715	0.831
CPN10	HSP91	0.088	0	0	0.221	0.184	0	0.740	0.829
RSR4	TRA2	0.184	0	0	0	0	0.800	0	0.829
MST1	OASA1	0.185	0	0	0.061	0	0.600	0.498	0.826
HSP91	MTHSC70-2	0	0.412	0.709	0.472	0.184	0.480	0.662	0.824
CLPB3	CR88	0.073	0	0	0.283	0.069	0	0.744	0.821
DHAR3	GSTU19	0	0.405	0	0	0	0.650	0.192	0.817
AT2G22360	CR88	0	0	0	0.274	0.152	0.251	0.646	0.815
NDPK1	NDPK2	0	0.449	0.949	0.057	0	0.800	0.847	0.815
CAT	NDPK1	0	0	0	0	0.598	0	0.540	0.807
AT5G14590	cICDH	0	0.449	0.979	0	0	0.800	0.810	0.805

GPX7	PRXQ	0.062	0	0	0	0	0	0.800	0.805
CLPB3	MTHSC70-2	0.058	0	0	0.474	0.183	0	0.569	0.802
AT3G60750	RSR4	0	0	0	0.050	0	0.800	0	0.801
CPN10	MTHSC70-2	0.088	0	0	0.339	0.278	0	0.595	0.800
AT5G63680	TRA2	0.178	0	0	0.167	0	0.600	0.330	0.792
AT2G22360	Hsp70-15	0.042	0.362	0	0.189	0.303	0.254	0.343	0.791
NDPK1	TRA2	0	0	0	0.053	0.759	0	0.141	0.786
NDPK2	TRA2	0	0	0	0.053	0.759	0	0.141	0.786
GOX1	SHM1	0.175	0	0	0.461	0	0	0.552	0.783
CAT	cICDH	0	0	0	0.082	0.080	0.649	0.353	0.782
AT1G53540	HSP21	0	0.444	0.763	0.709	0	0	0.708	0.781
AT2G22360	HSP91	0.042	0.363	0	0.134	0.303	0.254	0.343	0.777
AT5G14590	MSD1	0.071	0	0	0.071	0.094	0.625	0.330	0.767
AT3G52990	TRA2	0.178	0	0	0.066	0	0.600	0.330	0.766
CPN10	Hsp81.4	0	0	0	0.557	0.184	0	0.402	0.765
Hsp81.4	ROF1	0	0	0	0.477	0.182	0.459	0.106	0.765
CSD1	NDPK2	0	0	0	0.114	0.659	0	0.282	0.764
MSD1	cICDH	0.071	0	0	0.054	0.094	0.625	0.330	0.763
GPX7	GSTF9	0	0	0	0.053	0	0.650	0.332	0.759
CPN10	Hsp70-15	0.088	0	0	0.249	0.184	0	0.610	0.753
At5g06290	CSD1	0	0	0	0.231	0.233	0	0.615	0.753
AT5G14590	CAT	0	0	0	0.082	0.080	0.649	0.262	0.752
CSD1	TRA2	0	0	0	0.170	0.702	0	0.060	0.747
CAT	NDPK2	0	0	0	0	0.229	0	0.672	0.736
AOR	DHAR3	0	0	0	0.733	0	0	0	0.733
NDPK2	PMSR4	0.048	0	0	0.110	0.522	0.309	0.188	0.731
GPX7	GSTF10	0	0	0	0.053	0	0.650	0.249	0.729
Hsp70-15	ROF1	0	0	0	0.556	0.147	0.251	0.156	0.728
AT5G14590	GOX1	0	0	0	0.048	0	0.603	0.319	0.720
GOX1	cICDH	0	0	0	0.048	0	0.603	0.319	0.720
CAT	PRXQ	0	0	0	0.046	0	0	0.718	0.719
DHAR3	GSTF9	0	0	0	0	0	0.650	0.230	0.719
CSD1	GPX7	0.057	0	0	0.105	0.185	0.332	0.481	0.718
GPX7	MSD1	0	0	0	0	0.256	0.371	0.433	0.711
NDPK1	PMSR4	0.048	0	0	0	0.522	0.309	0.188	0.710

CLPB3	Hsp81.4	0.073	0	0	0.263	0.069	0	0.592	0.706
CLPB3	CPN10	0.071	0	0	0.164	0	0	0.651	0.706
PMSR4	PRXQ	0.190	0	0	0.206	0	0	0.578	0.705
